# A Randomized Controlled Study Comparing the Efficacy of 75mg Versus 150mg Aspirin for the Prevention of Preeclampsia in High-Risk Pregnant Women

**DOI:** 10.7759/cureus.39752

**Published:** 2023-05-30

**Authors:** Nishi Sinha, Shruti Singh, Mukta Agarwal, Pramod K Manjhi, Rajesh Kumar, Sunil Kumar Singh, Aakanksha Priya

**Affiliations:** 1 Pharmacology, All India Institute of Medical Sciences Patna, Patna, IND; 2 Obstetrics and Gynecology, All India Institute of Medical Sciences Patna, Patna, IND

**Keywords:** randomized control trial, primary outcome, preeclampsia prevention, low dose aspirin, fetomaternal outcome

## Abstract

Background

Preeclampsia is a major factor in both maternal and fetal morbidity and mortality. The most widely investigated preeclampsia prevention medication is low dose Aspirin. However, guidelines differ considerably regarding the prophylactic dose of Aspirin for preeclampsia.

Objective

The objective is to compare the efficacy of 150mg versus 75mg Aspirin for the prevention of preeclampsia in pregnant women at high risk of preeclampsia.

Methodology

This was a parallel, open-label, randomized control trial carried over a period of one year and three months at a tertiary care center of Eastern India. Block randomization was done and block sizes of 2 and 4 were used to ensure balanced distributions within the study arms. Primary outcome was the development of preeclampsia and secondary outcomes were fetomaternal complications in both groups.

Results

The present clinical trial was conducted on 116 pregnant women with a risk factor of preeclampsia and they were randomly assigned to receive either 150mg or 75mg of Aspirin daily beginning from 12 to 16 weeks of gestation till 36 weeks' gestation. A significantly greater number of pregnant females who received Aspirin 75mg (33.92%) developed preeclampsia in contrast to those who received Aspirin 150mg (8.77%), p=0.001, OR = 5.341, 95%CI = 1.829-15.594. There was an insignificant difference in fetomaternal outcome among both the groups of women.

Conclusion

Among women who are at high risk of developing preeclampsia, Aspirin 150 mg once a day at bedtime is more effective than Aspirin 75 mg once a day at bedtime in preventing preeclampsia with similar fetomaternal outcomes (NICU admission, IUGR, neonatal death, still birth, eclampsia, HELLP syndrome, placental abruption and pulmonary edema).

## Introduction

Hypertensive disease during pregnancy is the single largest cause of adverse pregnancy outcomes, complicating 5% to 10% of all pregnancies and leading to significant maternal and infant morbidity and mortality [1]. Hypertensive diseases during pregnancy include preeclampsia, pregnancy-induced hypertension, chronic hypertension preceding pregnancy, chronic hypertension with superimposed preeclampsia, and eclampsia. Out of all these related hypertensive disorders, preeclampsia syndrome, either alone or superimposed on chronic hypertension, is the most dangerous complicating 2% to 3% of pregnancies worldwide [2].

Preeclampsia is associated with several maternal and neonatal complications. Acute maternal complications include eclampsia, stroke, disseminated intravascular complication, Hemolysis, Elevated Liver enzyme, Low Platelet count syndrome (HELLP), pulmonary edema, adult respiratory distress syndrome, acute renal failure, and death. The long-term complications include chronic hypertension, diabetes mellitus, chronic renal failure, and coronary artery disease [3]. Neonates born to women with preeclampsia are at increased risk of NICU admission with low Apgar scores, intrauterine death (IUD), low birth weight, and intrauterine growth restriction (IUGR)[4].

Maternal endothelium damage is a major contributor to preeclampsia. Endothelial damage in preeclampsia reduces vascular endothelium production of prostacyclin (PGI_2_), a strong vasodilator and platelet aggregation inhibitor. Vascular endothelium injury exposes subendothelial collagen, which can cause platelet aggregation, stimulation, and leak of platelet-derived thromboxane A2 (TxA_2_), a strong vasoconstrictor and platelet aggregation stimulator. The reversal of the normal ratio of PGI_2_ and TxA_2_ observed in preeclampsia may be due to decreased PGI_2_ production by dysfunctional endothelial cells and increased TxA_2_ released by activated platelets and trophoblast. Considering the seriously adverse pregnancy outcomes associated with preeclampsia, an aggressive preventive approach is advisable. A variety of drugs including low-dose Aspirin (LDA), heparin, calcium supplementation, and antioxidants have a proven role in the prevention and treatment of preeclampsia. However, LDA has been associated with the most promising effect in preeclampsia.

LDA (60mg to 150mg) is a preventive agent for preeclampsia as it inhibits TxA_2_ synthesis, which results in less platelet aggregation and thus decreases placental ischemia [5]. LDA decreases the risk of preeclampsia by about one-quarter and premature delivery by about 14%, without an increased risk of bleeding in fetuses or newborn babies. There is also no increased risk of premature separation of the placenta or maternal bleeding [6]. Over the years, numerous studies have been done researching the timing of starting LDA therapy and duration of Aspirin in pregnant females for the prevention of preeclampsia, but with conflicting results. The dosage of Aspirin prophylaxis for preeclampsia is also heavily debated. In a study by Duley et al., LDA (range 60 to 150 mg/day) reduced the risk of preeclampsia by 24% and reduced the risk of preterm birth by 14%, and IUGR by 20%[7].

In another study by Talari, there was a significant difference between the Aspirin 80mg and placebo groups in the incidence of preeclampsia (2.5% versus 22.5%) [8]. Rolnik et al. conducted a study of Aspirin 150mg versus placebo and observed that the incidence of preeclampsia in Aspirin treated patients was significantly less as compared to placebo (1.6% versus 4.3%) (OR = 0.38, 95% CI= 0.20-0.74) [9].

It is noteworthy that Aspirin has been consistently used over the years for the prevention of preeclampsia in high-risk pregnant females, and there is no paucity of research on the use of Aspirin 75mg/day to 150mg/day versus placebo for the prevention of preeclampsia. However, studies with a head-to-head comparison of the efficacy of Aspirin 75mg/day versus 150mg/day are majorly lacking. Therefore, the present study compared the efficacy of Aspirin 150mg/day and 75mg/day, in high-risk pregnant females for the prevention of preeclampsia.

## Materials and methods

Study design

This was a parallel, open-label randomized controlled trial conducted in the Department of Pharmacology in collaboration with the Department of Obstetrics and Gynaecology of a tertiary care center in Eastern India. The study population comprised pregnant women between 12 and 16 weeks of gestational age enrolled between January 2021 and March 2022. They were recruited from the obstetrics outpatient department (OPD) and followed up till the end of puerperium (six weeks post-delivery). Ethical clearance was obtained from the Institutional Ethical Committee (Ref. no. AIIMS/Pat/IEC/PGTh/Jan20/03).

Sample size calculation

The estimate of the sample size was made using data that had been combined from a prior study [10], with a two-sided confidence interval of 95% and an 80% power assumption. The open epi software version 3 was used to calculate the sample size. The final sample size, taking into account the anticipated dropouts (presuming 20%), was 116.

Study population

Pregnant women (age ≥18 and <40 years, singleton or multifetal gestation, and live fetus at 12-16 weeks) with a high risk of preeclampsia were included in the study. Risk factors of preeclampsia based on clinical risk factors are as follows: (a) at least one high-risk factor- history of preeclampsia in a previous pregnancy, DM (type 1 or 2), chronic hypertension (systolic blood pressure (SBP) 140 mmHg or diastolic blood pressure (DBP) 90 mmHg which is diagnosed before pregnancy or before 20 weeks' gestation.), autoimmune disease, (b) at least two intermediate-risk factors- BMI ≥ 28 kg/m^2^, advanced maternal age of ≥35 years, family history of preeclampsia, nulliparity. Pregnant women with known Aspirin allergy, history of asthma, diagnosed peptic ulcer, and known liver disease, renal disease, and bleeding disorder were excluded from the study [11]. The potential study population was given written information about the trial and explained all aspects of the study. They were motivated to enroll in the study and those who agreed to participate provided written informed consent. Participants were clearly informed that they had the freedom to withdraw from the study, without loss of care at any stage.

Randomization details

Included pregnant women were randomly assigned into two groups. Group A received Aspirin 75mg once daily at bedtime and Group B received Aspirin 150mg once daily at bedtime. Block randomization was done with the help of an online site; http://www.sealedenvelope.com. Block sizes 2 and 4 were used to ensure balanced distributions within the study arm. Allocation was done by sequentially numbered opaque sealed envelopes (SNOSE). A unique code was written in the SNOSE and then participants were asked to pick an envelope in the order of enrolment and the corresponding dose of Aspirin was given.

Data collection

At the time of enrollment, demographic characteristics such as maternal age, height, weight, gestational age, and obstetrical history were obtained. Vitals such as blood pressure (BP) and pulse rate are recorded. Lab parameters such as complete blood count, blood grouping, and urine protein by dipstick method were obtained. Follow-up visits were done at intervals of two months, at delivery, and at four to six weeks postpartum. Pregnant women were advised to measure their BP at home. During follow-up, if BP of pregnant women was found to be raised (≥ 140/90 mmHg) then serum creatinine, serum ALT, platelet count, and fundus examination were advised. At each follow-up visit until delivery, pregnant women were questioned about the use of any medications since their last visit, and the study instructions were repeated. Weight and BP were measured, and urine protein by dipstick method was also investigated at each follow-up. Participants with raised BP (≥ 140/90 mm of Hg) antihypertensive medications were prescribed. Fetomaternal complications were recorded till six weeks post-delivery.

Outcomes of study

The primary outcome was the development of preeclampsia. Preeclampsia in the susceptible population was diagnosed by the development of gestational hypertension (BP ≥ 140/90 mm Hg) with proteinuria (urine protein 1+ by dipstick method) during the second half of pregnancy (≥20 weeks). Secondary outcomes were a comparison of fetomaternal outcomes at two varying doses.

Statistical analysis

Categorical outcomes (risk factors of preeclampsia, aspirin dose, SBP, and DBP Category) were compared among the study groups using the Chi-square test. Comparison of quantitative variables (mean age, mean BMI, mean gestational age, change in SBP, DBP, and urine protein) between the treatment groups was done by independent sample t-test. Univariate and multivariate logistic regression models were used to estimate the crude OR (cOR) and adjusted OR (aOR) for the association between potential risk factors and preeclampsia. p-value <0.05 was considered significant. Data were analyzed with the help of International Business Machines Corporation (IBM) Statistical Package for the Social Sciences (SPSS) version 20.0 and Microsoft Excel.

## Results

**Figure 1:**
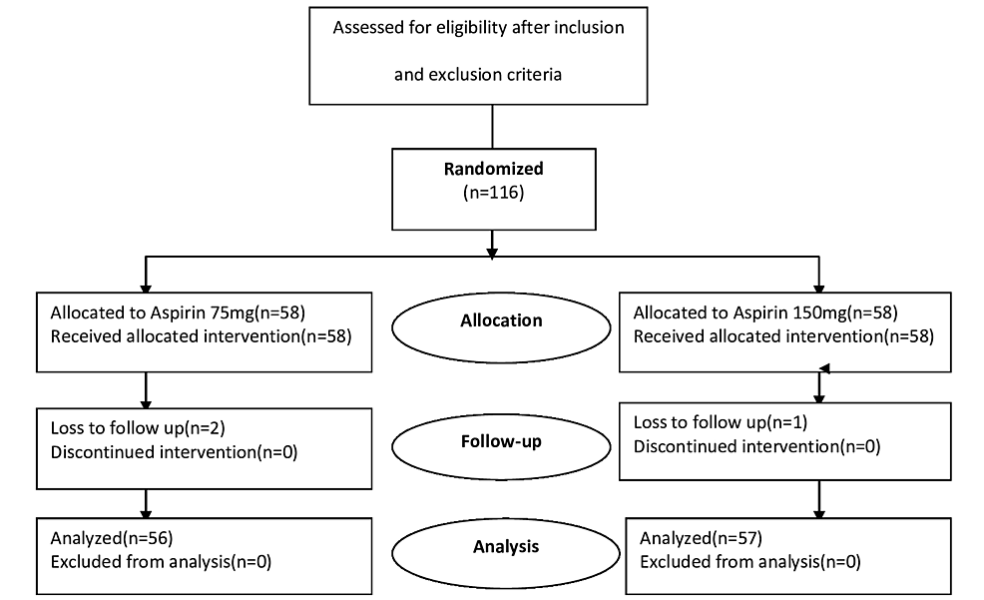
CONSORT flowchart of randomized controlled trial

A total of 116 patients were enrolled in the study; 58 patients were given Aspirin at a dose of 75mg and the rest were given Aspirin at a dose of 150mg, for the prevention of preeclampsia. Out of 116 patients, three patients were lost to follow-up. Figure 1 shows the CONSORT flowchart of this randomized controlled trial.

Table 1 presents a summary of the participants demographic and obstetrical characteristics. The mean age of pregnant women in group A was 28.05±06.19 years and group B was 26.84±05.21 years. The mean BMI of pregnant women in group A was 25.38±3.00 kg/m² and group B was 25.82±3.16 kg/m². The mean gestational age of pregnant women in group A was 14.10±1.24 years and group B was 14.42±1.16 years. The distribution of all risk factors (preeclampsia in previous pregnancy, family history of preeclampsia, obesity, diabetes mellitus, chronic hypertension, autoimmune disease, advanced maternal age) across the treatment groups was comparable.

**Table 1:** Characteristic of included pregnant women in the study (N=113) Group A=75mg, Group B=150mg

Variable	Study groups		Total (N=113)	Chi-square value	P-value
	Group A (56)	Group B (57)			
Age (mean ± SD)	28.05±06.19	26.84±05.21			0.259
BMI (mean±SD)	25.38±3.00	25.82±3.16			0.448
Gestational Age (mean±SD)	14.10±1.24	14.42±1.16			0.169
Preeclampsia in previous pregnancy	13(23.21%)	15(26.32%)	28(24.8%)	0.146	0.703
Family history of preeclampsia	11(19.64%)	16(28.07%)	27(23.89%)	1.103	0.294
Obesity (>28kg/m²)	11(19.64%)	15(26.32%)	26(23.00%)	0.71	0.399
Diabetes Mellitus	11(19.64%)	13(22.80%)	24(21.24%)	0.169	0.681
Chronic Hypertension	23(41.10%)	29(50.88%)	52(46.00%)	1.093	0.296
Autoimmune disease	2(3.51%)	1(1.75%)	3(2.65%)	0.361	0.548
Advanced maternal age >35 years	13 (23.21%)	7(12.28%)	20(17.70%)	2.318	0.128

The incidence of preeclampsia was assessed across Aspirin doses (75mg versus 150mg), history of chronic hypertension, baseline SBP, mean SBP category (mean of SBP at each follow up), baseline DBP and mean DBP category (mean of DBP at each follow-up). A significantly greater number of pregnant females who received Aspirin 75mg (33.92%) developed preeclampsia in contrast to those who received Aspirin 150mg (8.77%) (χ²=10.687, p= 0.001). There were five times greater odds of preeclampsia in those who received Aspirin 75mg in comparison to 150mg (cOR= 5.341, CI= 1.829-15.594). There was a statistically significant difference in the incidence of preeclampsia on the basis of chronic hypertension OR (95% CI) = 4.853(1.753-13.432), Baseline SBP OR (95% CI) = 3.508(1.272-9.673), mean SBP OR (95% CI) = 7.02(2.296-21.77) and mean DBP OR (95% CI) = 5.83(2.197-15.47) (Table 2).

**Table 2:** The incidence of preeclampsia between two groups according to Aspirin doses and maternal risk factors at enrollment (N=113) Group A=75mg, Group B=150mg

Variables	Preeclampsia		Chi-square value	P-value	cOR (CI)
	Yes	No			
Study Groups					
Group A (56)	19(33.92%)	37(66.07%)	10.687	0.001*	5.341(1.829-15.594)*
Group B (57)	05(8.77%)	52(91.22%)			1
Chronic hypertension					
Present	18(34.60%)	34(65.40%)	10.304	0.001*	4.853(1.753-13.432)*
Absent	06 (9.80%)	55(90.2%)			1
Baseline SBP					
≤140 mm of Hg	15(16.5%)	76(83.5%)	6.319	0.012*	1
≥140 mm of Hg	09(40.9%)	13(59.1%)			3.508(1.272-9.673)*
Baseline DBP					
≤90 mm of Hg	14(20.6%)	54(79.4%)	0.043	0.835	1
≥90 mm of Hg	10(22.2%)	35(77.8%)			2.166(0.858-5.471)
Mean SBP					
≤140 mm of Hg	15(15.46%)	82(84.53%)	13.66	<0.001*	1
≥140 mm of Hg	09(56.25%)	07(43.75%)			7.02(2.296-21.77)*
Mean DBP					
≤90 mm of Hg	11(12.94%)	74(87.05%)	14.12	<0.001*	1
≥90 mm of Hg	13(46.42%)	15(53.57%)			5.83(2.197-15.47)*

A multiple logistic regression analysis was conducted for those variables that significantly (95% CI) influenced risks of preeclampsia in the univariate analyses (Aspirin dose, chronic hypertension, baseline SBP, mean SBP and mean DBP). The logistic regression model was statistically significant, χ2 (6) = 12.193, p=0.058. The model explained 47% (Nagelkerke R2) of the variance in preeclampsia. Results of the final model of logistic regression showed that the odds of preeclampsia in those who received aspirin 75mg was significantly higher in contrast to those who received 150mg (aOR=9.060, 95% CI=2.334-35.169) after adjustment for other significant variables. Except baseline SBP, Chronic hypertension 5.884(1.377-25.141), mean SBP 6.720(1.449-31.167) and mean DBP 5.019(1.479-17.033) remained significantly associated with incidence of preeclampsia after adjustment for other significant variables (Table 3).

**Table 3:** Results of multivariate logistic regression for association between Aspirin doses and preeclampsia after adjustment for maternal risk factors (N=113)

Variables	cOR(95%CI)	aOR(95%CI)	P-value
Aspirin doses (75mg and 150mg)	5.341(1.82-15.59)*	9.060(2.334-35.169)*	0.001*
Chronic hypertension	7.086(2.12-23.68)*	5.884(1.377-25.141)*	0.017*
Baseline SBP	3.508(1.28-9.67)*	1.828(0.416-8.037)	0.425
Mean SBP	7.029(2.29-21.77)*	6.720(1.449-31.167)*	0.015*
Mean DBP	5.83(2.19-15.47)*	5.019(1.479-17.033)*	0.010*

An independent sample t-test was conducted to look for any significant difference in change in various objective vital parameters (SBP, DBP, Urine protein) of the two study groups, from enrollment till delivery. There was a statistically insignificant difference between the two study groups with reference to change in SBP, DBP from first enrollment till delivery. The mean urine protein in group A was 0.48±0.78 and group B was 0.14±0.48 at delivery. Urine protein was investigated with the help of dipstick method. A significantly greater increase in urine protein was observed in Group A in comparison to Group B (t=2.796, p=0.006) (Table 4).

**Table 4:** Comparison in terms of Vital signs at enrollment and visit to hospital for childbirth between treatment groups (N=113) Group A=75mg, Group B=150mg

Variables	Group A (56)	Group B (57)	t-value	P-value
SBP at enrollment	132.08±6.18	133.00±4.73		
SBP at delivery	125.53±13.52	128.21±17.91		
SBP difference during the study period	6.55±13.93	4.78±17.98	0.582	0.562
DBP at enrollment	87.19±7.76	89.49±6.71		
DBP at delivery	85.23±9.52	84.03±5.73		
DBP difference during the study period	1.96±10.67	5.45±8.28	-1.94	0.06
Urine protein at onset of study	0	0		
Urine protein at delivery	0.48±0.78	0.14±0.48		
Urine protein difference during the study period	0.48±0.78	0.14±0.48	2.796	0.006*

**Figure 2:**
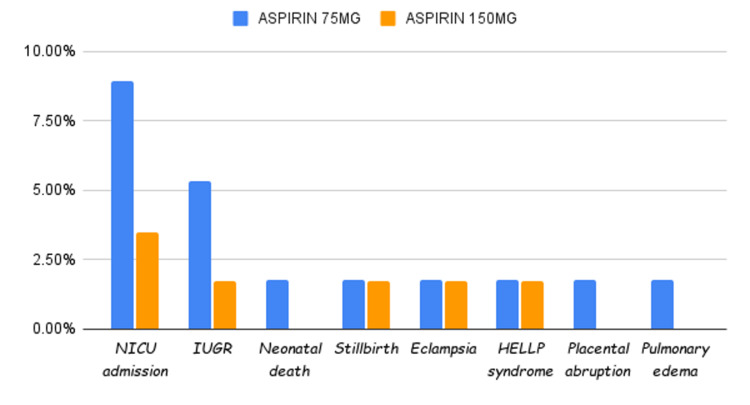
Comparison of fetomaternal complications between the study groups (N=113)

Figure 2 shows the fetomaternal outcome of both groups. NICU admission, IUGR, neonatal death, still birth, eclampsia, HELLP syndrome, placental abruption and pulmonary edema were the observed fetomaternal outcomes. There was a statistically insignificant difference in the fetomaternal outcome of the two study groups.

## Discussion

Preeclampsia is known as a disease of theories. Its etiology is unknown, making it difficult to determine how to best prevent it. Its prevention is critical due to the complications it can cause. LDA appears to be the most promising method of prevention, but it is also fraught with controversy regarding its efficacy, dosage, and safety. Considering the dose-dependent toxicity of aspirin, it is vital to understand the dosage that offers an acceptable risk-benefit ratio.

In 1979, a study found that women who took Aspirin regularly during pregnancy were less likely to develop preeclampsia than women who did not [9]. More than 30 trials were conducted in the subsequent decades to investigate the benefit of LDA (at a dose of 50 to 150mg per day) for the prevention of preeclampsia; a meta-analysis of these studies revealed that such therapy resulted in a 10% lower incidence of preeclampsia [12]. A recent meta-analysis concluded that starting LDA at or before 16 weeks was associated with a 50% reduction in overall preeclampsia risk [13,14].

However, the dose of Aspirin for preeclampsia prophylaxis has remained controversial with studies showing varying results with Aspirin 75mg to 150mg. The current study compared the efficacy of Aspirin at two varying doses (75mg and 150mg) for the prevention of preeclampsia in pregnant women.

The distribution of preeclampsia found in the Aspirin 75mg treated and Aspirin 150mg treated groups in this study were 33.92% and 8.77%, respectively. There was a significant group difference in the incidence of preeclampsia (p=0.001) across the treatment groups. Aspirin at a dose of 75mg had a higher rate of preeclampsia compared to Aspirin 150mg (OR=5.341, 95 % CI= 1.829-15.594); a finding similar to the study of Kumar et al. [15]. They found a significant difference (p= 0.046) in the incidence of preeclampsia with an Aspirin dose of 75mg versus 150mg (17.2% versus 6.5%). Roberge [16], in a meta-analysis of 45 RCTs comparing the effect of daily Aspirin in various doses or placebo during pregnancy, found that prevention of preeclampsia and fetal growth restriction using Aspirin in early pregnancy is associated with a dose-response effect (50-150mg), with higher dosage having a better effect on prevention of preeclampsia (p < 0.001), severe preeclampsia (p = 0.008) and fetal growth restriction (p < 0.001), while LDA initiated at more than 16 weeks gestation has a modest or no impact on the risk of preeclampsia, severe preeclampsia, and fetal growth restriction. Rolnik et al. [9] conducted a study of Aspirin 150mg versus placebo in high-risk pregnancies for the prevention of preeclampsia and observed that the incidence of preeclampsia in Aspirin treated patients was significantly less as compared to placebo (1.6% versus 4.3%) (OR= 0.38, 95 % CI= 0.20-0.74). In contrast, Obido et al. [17] conducted a study of Aspirin 81mg versus placebo in high-risk pregnancies and concluded that the incidence of preeclampsia in Aspirin treated patients as well as placebo-treated patients was the same (20%). This was possibly due to a significant dropout rate in this trial. A similar study done by Liu et al. [18] observed that Aspirin 50mg, 75mg, and 100mg had comparable effects on the incidence of preeclampsia (p=0.935).

Urinary protein is a sign of early hypertensive kidney lesion and is a major cause of disease progression as in the case of preeclampsia. The degree of blood pressure is positively associated with different urine protein levels. In our study, urine protein increased significantly in Aspirin 75mg treated groups (0.48±0.78) as compared to Aspirin 150mg treated groups (0.14±0.48) (p=0.006). At the enrollment, urine protein was 0 by the dipstick method. Aspirin 150mg showed a lesser increment of urine protein as compared to the 75mg treated groups. This finding is in concordance with those of Liu et al. [18], who observed statistical differences in urine protein in aspirin 75mg and 100mg treated groups (p=0.039 and 0.037, respectively).

In this study, mean SBP showed a decreasing trend from 132.12±6.18 to 125.53±13.52 in Group A while 133.00±4.73 to 128.21±17.91 in Group B. Also, mean DBP decreased from 87.19±7.76 to 85.23±9.52 in Group A while 89.49±6.71 to 84.03±5.73 in Group B (Table 4). However, the decrease in mean SBP and DBP were not significantly different in both groups. A similar finding is consistent with that of Liu et al. [18], who observed no statistical difference in mean arterial pressure (MAP) in 50mg, 75mg, and 100mg Aspirin treated groups (p=0.421). In their study, Aspirin 75mg decreased MAP from 114.4±18.2 to 85.8±12.7 similar to that of Aspirin 100mg which decreased MAP from 109.2±20.2 to 86.6±16.9 mmHg. In contrast, a study done by Abdi et al. [19] showed that Aspirin 80mg increased SBP by 8.25±14.83 mmHg and increased DBP by 6.92±11.46 mmHg.

The incidence of NICU admission, IUGR, and neonatal death was lower in women given 150mg Aspirin although the difference was not statistically significant. Another fetal outcome like stillbirth had an equal incidence in Aspirin 75mg as well as 150mg Aspirin. This finding is similar to Yu et al. [20] and Hoffman et al. [21], who compared Aspirin (150mg and 81mg, respectively) versus placebo and observed no statistical significance in the incidence of NICU admission, death, and stillbirth. Lin et al. [22] also found no statistical significance difference in the incidence of death and stillbirth. However, a contrary finding was observed by USPSTF, 2014. USPSTF concluded that LDA had a beneficial effect on perinatal outcomes, IUGR was reduced by 20 %, and preterm birth was reduced by 14% [23].

Similar to the ASPRE trial, our trial showed that the incidence of preeclampsia in the 150mg Aspirin group was lower than that in the 75mg group; however, we also realized that in our study population, there were similar fetomaternal outcomes (such as NICU admission, IUGR, neonatal death, stillbirth, eclampsia, HELLP syndrome, placental abruption, and pulmonary edema) in both the groups.

The main limitation of the current study was the small sample size. We were also unable to collect data about the time at which preeclampsia occurred, in both groups. So, we could not compare the time at which beneficial effects are exerted by 150mg versus 75mg. This study did not extend monitoring of both participants and their babies beyond six weeks postpartum; so long-term effects of the drug after puerperium could not be determined.

## Conclusions

The study concludes that among women with high-risk pregnancies, the use of Aspirin 150mg per day at bedtime starting between 12 and 16 weeks of gestation till 36 weeks is a potent intervention to reduce the development of preeclampsia as compared to a dose of 75mg per day at bedtime. This study also showed that there is a similar fetomaternal outcome in both groups comparing 75mg and 150mg Aspirin (NICU admission, IUGR, neonatal death, stillbirth, eclampsia, HELLP syndrome, placental abruption, and pulmonary edema).
